# Development of a non‐lethal stomach content analysis method for freshwater eels: An empirical evaluation of the tube method for *Anguilla marmorata*

**DOI:** 10.1111/jfb.70198

**Published:** 2025-08-27

**Authors:** Tatsuhiko Maeda, Hikaru Itakura, Ryoshiro Wakiya, Shingo Kimura

**Affiliations:** ^1^ Atmosphere and Ocean Research Institute The University of Tokyo Chiba Japan

**Keywords:** Amami–Ohshima Island, Anguilliformes, feeding ecology, non‐lethal stomach content analysis, yellow eel

## Abstract

Understanding the feeding habits of predatory fish is essential for unravelling food web structures and implementing conservation strategies in riverine ecosystems. However, conventional lethal stomach content analysis methods are not necessarily appropriate for long‐term dietary studies, particularly for threatened species, as they require large sample sizes due to the inability to repeatedly analyse stomach contents from the same individuals. This study aimed to develop and validate a non‐lethal stomach content analysis method using tubes for the giant mottled eel *Anguilla marmorata*, a representative tropical anguillid species. A total of 205 eels were collected from nine rivers on Amami–Ohshima Island, Japan. Among 102 eels, including stomach contents, the tube method achieved an average removal efficiency of 76.5% (biomass content) and a detection rate of 92.4% for prey categories, effectively capturing dietary composition without significant bias. The most important food items were prawns (mainly *Macrobrachium*), crabs and fish, but aquatic insects, terrestrial invertebrates and a few snails were also eaten. Although crabs were less efficiently removed due to their body size or hard exoskeletons, supplementary use of forceps allowed complete collection of their stomach contents. The removal efficiency was not significantly influenced by eel size, stomach fullness or prey type, suggesting broad applicability of this method. Our findings demonstrate that the tube method, combined with forceps when necessary, offers a powerful non‐lethal tool for investigating individual‐level feeding ecology of anguillid eels, enabling long‐term dietary monitoring and supporting conservation of declining populations. This method will advance ecological understanding and sustainable management of anguillid eels and their freshwater habitats, and this is especially true for tropical eels whose feeding ecology has rarely been studied.

## 1 INTRODUCTION

Biodiversity conservation requires precise information on the food web structure, the species interactions and energy flows within ecosystems, all of which can be elucidated by studying the feeding habits of animals that constitute the ecosystem (Cardinale et al., 2012; Link, 2002). Particularly, feeding habits of top predators provide information on direct predator–prey interactions. Top predators often contribute to maintaining biodiversity in ecosystems by regulating the food web structures through predation (Hammerschlag et al., 2019; Letnic et al., 2012). These predators serve as indicators of ecosystem health, with their feeding habits reflecting prey availability and trophic shifts in food webs (Hazen et al., 2019). Therefore, understanding the feeding habits of top predators is crucial for the conservation of biodiversity.

Many studies investigating feeding habits of fishes rely on stomach content analysis using lethal methods, which involve collecting stomach contents by surgically opening the digestive tract after euthanasia (Manko, 2016). This approach shows direct information about the food items of each individual of the target species, but provides only a snapshot of feeding behaviour and is often biased by being based on the last feeding event (Baker et al., 2024). Moreover, as prey items consumed by fish exhibit spatiotemporal variability, the sample size required for an accurate assessment of stomach contents increases with prey diversity (Silveira et al., 2020). A larger sample size is needed for accurate dietary assessment for generalist predators who consume a wide range of prey organisms (Jellyman, 1989). Consequently, this method requires the collection of a large number of samples (Amundsen & Sánchez‐Hernández, 2019), making it challenging to conduct long‐term or spatiotemporally extensive investigations of fish diets. This is a particularly critical issue for monitoring the diets of endangered species, as the lethal method poses a risk of population decline (Thorstensen et al., 2022).

Stomach content analysis using non‐lethal methods facilitates the collection of samples from many individuals and has the potential to enable long‐term monitoring of fish diets (e.g., Kreiling et al., 2021). These methods provide detailed insights into individual‐level feeding ecology, including dietary specialisation or changes in trophic niche, and are related to growth rate, dietary shifts and the availability of prey (Condini et al., 2023; Elston et al., 2020; Natsumeda et al., 2012). Several non‐lethal methods have been developed to investigate stomach contents for fish species, such as using tubes, gastroscopes, stomach flushing or emetic chemicals causing regurgitation (Kamler & Pope, 2001). The tube method is widely used as a non‐lethal method for stomach content analysis (White, 1930). In this method, a tube is inserted through the oesophagus into the stomach, and pressure is applied to force the stomach contents into the tube for collection. The tube method is easy to implement in the field and does not require any other special equipment. Although the tube method has been used to examine the feeding habits of a variety of fish (Kamler & Pope, 2001; Myers et al., 2025), it has rarely been applied to fish species with small mouths and long stomachs relative to their body size (e.g., Anguilliformes), possibly due to lower removal efficiency (Van Den Avyle & Roussel, 1980). As the efficiency of the tube method varies depending on the fish species, fish size and prey type (Kamler & Pope, 2001), it is necessary to evaluate the effectiveness of this non‐lethal method for each target fish species before applying it in dietary studies.

Anguillid eels (genus *Anguilla*) have catadromous life cycles, in which they spawn in the open ocean and grow in continental waters such as rivers and estuaries (Tesch, 1977), where they spent several years or decades during their growth phase as yellow eels (Hagihara et al., 2018; Jessop, 2010; Todd, 1980). They are well known as opportunistic generalist predators, feeding on a wide range of species, including aquatic and terrestrial organisms, whose availability potentially varies greatly depending on seasons and habitats (Denis et al., 2022; Itakura et al., 2021; Nishimoto et al., 2023; Wakiya & Mochioka, 2021). In addition, the rate of vacuity (empty stomachs) of eels is often high, ranging from 30% to 70% (Itakura et al., 2015; Jellyman, 1989; Kaifu, Miyazaki, et al., 2013), indicating that a large number of samples (e.g., several hundred individuals or more) are required to accurately assess their diets (Itakura et al., 2015; Kaifu, Miyazaki, et al., 2013). Consequently, long‐term, high‐frequency dietary monitoring of anguillid eels is lacking because of the reliance on lethal sampling methods such as dissection. The development of non‐lethal methods for stomach content analysis in anguillid eels will advance our understanding of their feeding ecology and support the conservation of their populations. Although one study has assessed the removal efficiency of a non‐lethal stomach content analysis method for the American eel (*Anguilla rostrata*) using gastric lavage (Studio & May, 2025), detailed information on removal efficiency, such as for prey types, remains unknown. The target species of the present study is one of the tropical eels, the giant mottled eel (*Anguilla marmorata*), which is the most geographically widespread anguillid eel in the world, occurring from the western Indian Ocean to the central South Pacific Ocean and north to southern Japan (IUCN, 2024). Compared to the Northern Hemisphere temperate eels, food habits of the tropical eels have been poorly studied, even though eels are ecologically important predatory species in river ecosystems (Itakura, Wakiya, Gollock, & Kaifu, 2020).

This study aimed (1) to develop an effective non‐lethal tube method for stomach content analysis in anguillid eels by evaluating its removal efficiency and (2) to elucidate the feeding ecology of *A. marmorata* through stomach content analysis. To this end, stomach contents of eels were collected using tubes followed by checking the existence of the remaining stomach contents by dissection. This study offers a validated, non‐lethal method for investigating dietary composition in *A. marmorata*, with potential applications for understanding the feeding ecology of tropical eels.

## 2 MATERIALS AND METHODS

### 2.1 Ethics statement

All experiments in this study, including capture and handling, were conducted in accordance with the principles of ethics of animal experiments at the University of Tokyo.

Sampling with electrofishing was reviewed and approved by the district government of Kagoshima Prefecture (permit number: 2006‐1).

### 2.2 Study area

**FIGURE 1:**
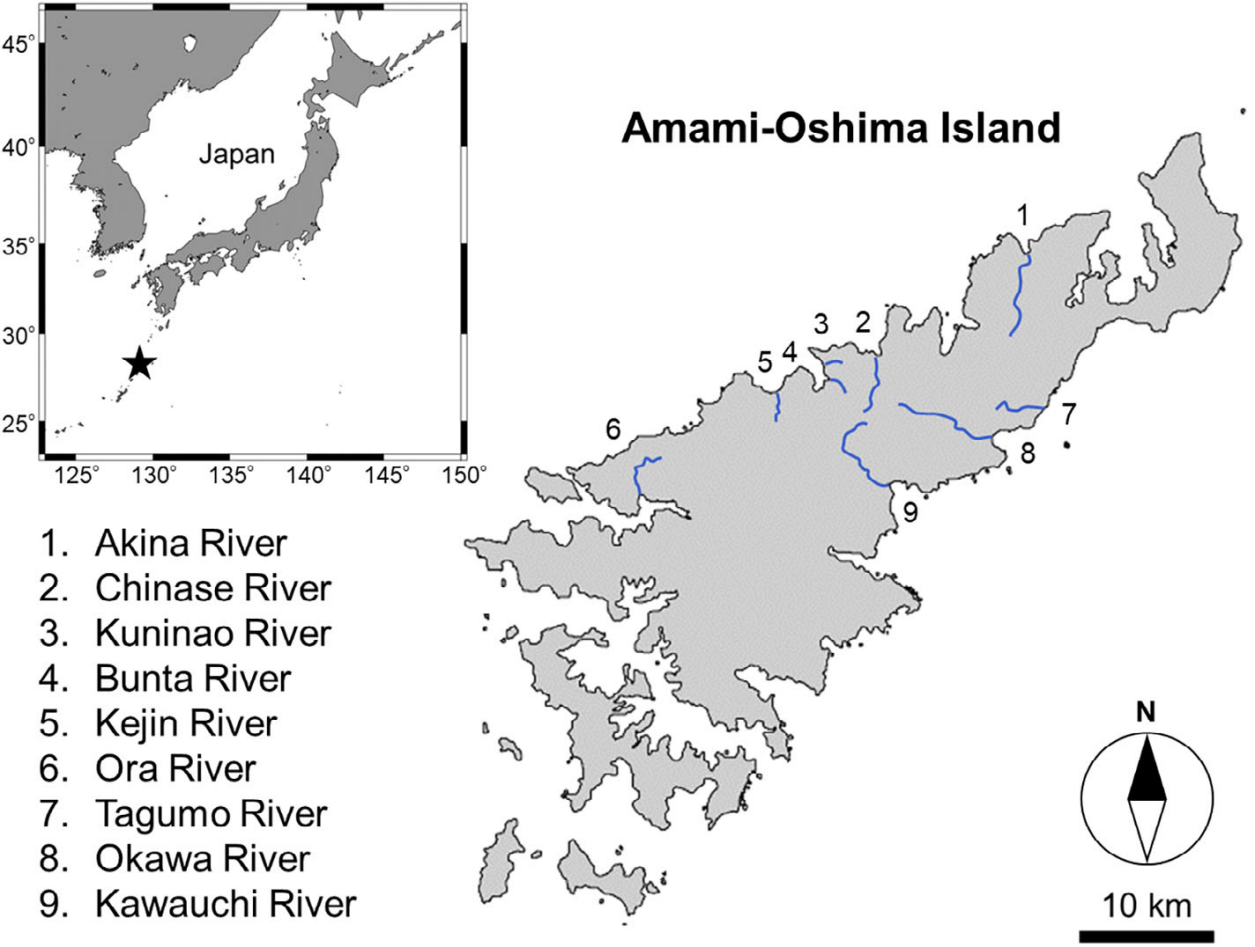
Map of the study area on Amami–Oshima Island, Kagoshima Prefecture, Japan. The star indicates the location of Amami–Oshima Island, and the numbers on the map indicate each river where *Anguilla marmorata* eels were collected.

This study was conducted in nine rivers on subtropical Amami–Oshima Island, Japan (Figure 1). This island is located between the southern mainland of Japan and the Okinawa Islands adjacent to the western North Pacific Ocean and on the eastern side of the Kuroshio Current, which is one of the strongest western boundary currents. This is the second‐largest island in the Nansei Islands (Okinawa–Jima Island is the largest) in terms of area. Amami–Oshima Island is relatively close to the northern limit of the distribution range of *A. marmorata* (IUCN, 2024), and *A. marmorata* is clearly the dominant anguillid species throughout the rivers on this island (Itakura & Wakiya, 2020; Itakura, Wakiya, Sakata, et al., 2020; Maeda et al., 2024; Wakiya et al., 2019).

### 2.3 Eel collection

Eel sampling surveys were carried out in freshwater areas that were 50–1500 m away from the river mouth in each river, where most sampling sites had gravel substrates and natural riverbanks. Eels were captured by a single person using a backpack electrofisher (150 V pulsed DC, LR‐24, Smith‐Root, Vancouver, Washington, USA) from August 2023 to July 2024. Because eels generally forage at night (Itakura et al., 2018; Kaifu, Miller, et al., 2013; Tesch, 1977), sampling was performed during the morning to avoid underestimating the easily digestible prey organisms. Captured eels were held in mesh bags in river water, where they were kept in the shade until they were anaesthetised using a 0.2‰ eugenol solution (FA100, DS Pharma Animal Health Co., Ltd., Japan) that was diluted with river water. Temperatures on the island may vary from 14°C (February) to 26°C (August) based on water temperature measurement from another river (Maeda et al., unpublished data). Once the eels were confirmed to have lost equilibrium, they were considered sufficiently anaesthetised and ready for the stomach flushing procedure, which occurred within 5 min of immersion for most individuals. Each specimen was identified morphologically following Watanabe et al. (2004), and their growth stage was confirmed based on the body and pectoral fin colouration following previous studies (Hagihara et al., 2012). A total of two sexually maturing individuals were excluded from this experiment, because they may not feed once they have already started their early migration to the ocean to spawn (Chow et al., 2010).

### 2.4 Non‐lethal stomach content analysis

#### 2.4.1 Tube method

**FIGURE 2:**
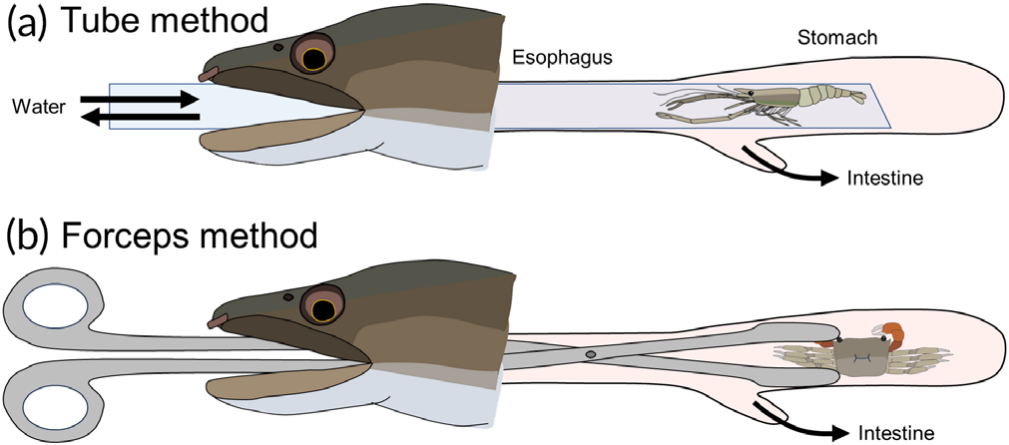
Diagrams of stomach content analysis procedures that were the tube and forceps methods. In the tube method (a), a tube is inserted from the mouth to the posterior end of the stomach, and stomach contents are aspirated by pumping water inward. The forceps method (b) involves inserting forceps orally and using them to directly grasp and retrieve the stomach contents.

The tube method was performed based on White (1930) with some minor modifications (Figure 2a; Video S1). After anaesthesia, a flexible transparent plastic tube, which was made from polyvinyl chloride resin, matching the gape size, was inserted through the mouth to the posterior end of the stomach. The eel was then laid down horizontally, and river water was poured in using a squeeze bottle. While holding the eel with its mouth facing downward and the stomach upward, stomach contents were collected by applying pressure to push water through the mouth into the tube, and then slowly pulling the tube out, as shown in Video S1. During the treatment, the hand holding the fish would massage the fish's abdomen to help it easily release the stomach contents (Hafs et al., 2011). This process was repeated at least thrice until the stomach contents could no longer be removed. Stomach contents were collected using six different tube diameters, which were selected based on the gape size of the captured fish following a previous study (Van Den Avyle & Roussel, 1980): inner and outer diameters (ID × OD) of 2 × 4 mm for individuals less than 275 mm in total length, 4 × 6 mm for those less than 400 mm, 8 × 10 mm for those less than 550 mm, 12 × 15 mm for those less than 730 mm, 15 × 20 mm for those less than 860 mm and 19 × 25 mm for those greater than 860 mm. Stomach contents were visually identified to the lowest possible taxonomic level using a Petri dish and tweezers. Prey items were divided into eight prey‐type categories: crabs, prawns, fish, snails, aquatic insect larvae, terrestrial invertebrates, plants and unidentified. The wet weight of food items found in each eel was measured to the nearest 0.1 g for each category.

After stomach content collection, captured eels were placed in mesh bags and submerged in each river to recover from the anaesthesia. All individuals fully recovered from anaesthesia immediately after being submerged in river water. They were then deeply anaesthetised with a > 1‰ eugenol solution for euthanasia before being stored at −20°C within 1 h. In the laboratory, the total length (mm) and body weight (g) of the eels were measured, and the stomach was opened by dissection to check for residual stomach content. The stomach was fully longitudinally incised and visually inspected for damage, such as punctures or bleeding. Residual stomach contents obtained through dissection were measured using the same methods as described above. For all eels, relative occurrence frequency was determined by the presence or absence of each prey category, and biomass proportion was calculated from its wet weight. The effectiveness of the tube method was evaluated by calculating the removal efficiency based on the biomass of food contents and the detection rate based on prey occurrences. Removal efficiency was calculated for each individual and prey category as the ratio of the stomach content weight obtained by the tube method to the total stomach content weight as follows (Bangley et al., 2013):

$$\text{Removal efficiency}\;\left(\%\right)=\left\{W_{T}/\left(W_{T}+W_{R}\right)\right\}\times 100$$

where *W*_*T*_ is the weight of stomach contents collected by the tube method, and *W*_*R*_ is the weight of residual stomach contents collected by dissection. Detection rate was calculated as the ratio of the number of occurrences of each prey category identified by the tube method to those identified only through dissection:

$$\text{Detection rate}\;\left(\%\right)=\left\{O_{T}/\left(O_{T}+O_{R}\right)\right\}\times 100$$

where *O*_*T*_ is the number of occurrences of prey categories by the tube method, and *O*_*R*_ is the number of occurrences of prey categories found only through dissection when not collected by the tube method. Stomach fullness was calculated using the method of Hyslop (1980):

$$\text{Stomach fullness}\;\left(\%\right)=\left\{\left(W_{T}+W_{R}\right)/\mathrm{BW}\right\}\times 100$$

where *W*_*T*_ is the weight of stomach contents collected by the tube method, *W*_*R*_ is the weight of residual stomach contents collected by dissection and BW is the body weight of eels. To describe the dietary composition of *A. marmorata*, frequency of occurrence percentage (F%) of each prey category was calculated based on the presence or absence of each prey category.

#### 2.4.2 Forceps method

As a result of stomach content analysis using the tube method and the subsequent dissection, some food items, such as large crustaceans, occasionally remain in the stomach, which cannot be aspirated by the tube (see the Results section). To improve the removal efficiency of the non‐lethal method, the forceps method (Wales, 1962) was used as a complementary approach to collect the remaining stomach contents after the tube method. To assess the removal efficiency of the forceps method, four additional individuals were collected in the Chinase River in October 2024, which were used for the analysis. Residual stomach content was recognised from all four individuals while feeling the fish abdomen by hand when the tube method was performed. After the tube method was performed, forceps were inserted through the mouth, and the stomach contents were extracted by directly grasping them with forceps (Figure 2b; Video S1). The removal efficiency was calculated for each individual as the ratio of the stomach content weights collected using both the tube and forceps methods to total stomach content weight following the aforementioned equation.

### 2.5 Data analysis

All statistical analyses were performed using the R statistical software (version 4.1.2; R Development Core Team 2021). A multimodality test was conducted to check the modality (unimodal or multimodal) of removal efficiency distribution using Silverman's test (*modetest* function of the *multimode* package). A generalised linear model (GLM), with a binomial distribution and a logit link function, was used to test whether the removal efficiency of the tube method was influenced by prey category, body size and the feeding condition of eels. The model included removal efficiency for each individual as a response variable and total length, prey category and stomach fullness as explanatory variables. When multiple prey categories were observed in the stomach content of a single individual, the category with the highest weight was selected as a representative prey category. To avoid multicollinearity of explanatory variables, the variance inflation factor (VIF) between total length and stomach fullness was calculated. This confirmed that the VIF included in the model was < 2, indicating no multicollinearity in the model (Marquardt, 1970). The *dredge* function of *the MuMIn* package was used for model selection, which generated models having all possible combinations of explanatory variables (Barton, 2024). The model with the lowest Akaike's Information Criterion (AIC) was selected as the best model. To evaluate whether diet composition differed between the tube method and all stomach contents (tube and dissection), we performed a permutational multivariate analysis of variance (PERMANOVA) using the *vegan* package. The Bray–Curtis dissimilarity index was used to calculate compositional dissimilarities, and 9999 permutations were applied to assess statistical significance.

## 3 RESULTS

### 3.1 Efficiency of non‐lethal stomach content analysis

#### 3.1.1 Effectiveness of tube method

**FIGURE 3:**
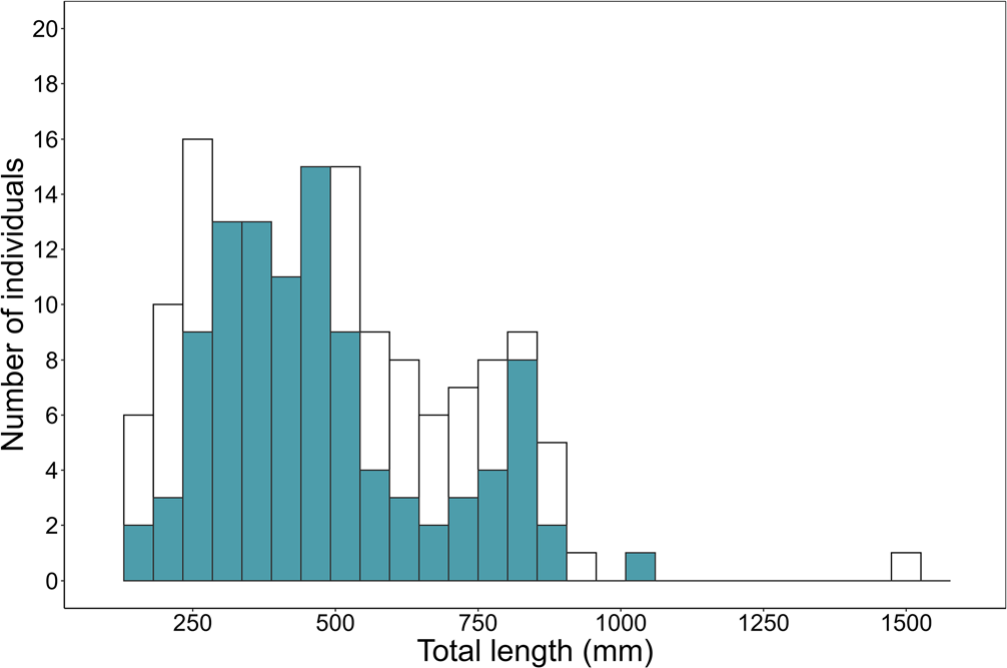
Histogram of the total length of the 204 *Anguilla marmorata* eels. Blue bars indicate individuals that had stomach contents and were used in this study, whereas white bars indicate individuals with empty stomachs.

A total of 204 individuals of *A. marmorata* were collected during the study period, and 102 of those eels had empty stomachs and were excluded from the analysis. On each sampling occasion, 4 to 22 eels were captured, and the number of individuals with stomach contents ranged from 0 to 15 individuals (Table S1). The total length and body weight [mean ± standard deviation (SD)] of the 102 individuals were 472.4 ± 192.1 mm (Figure 3) and 410.9 ± 533.5 g, respectively.

**FIGURE 4:**
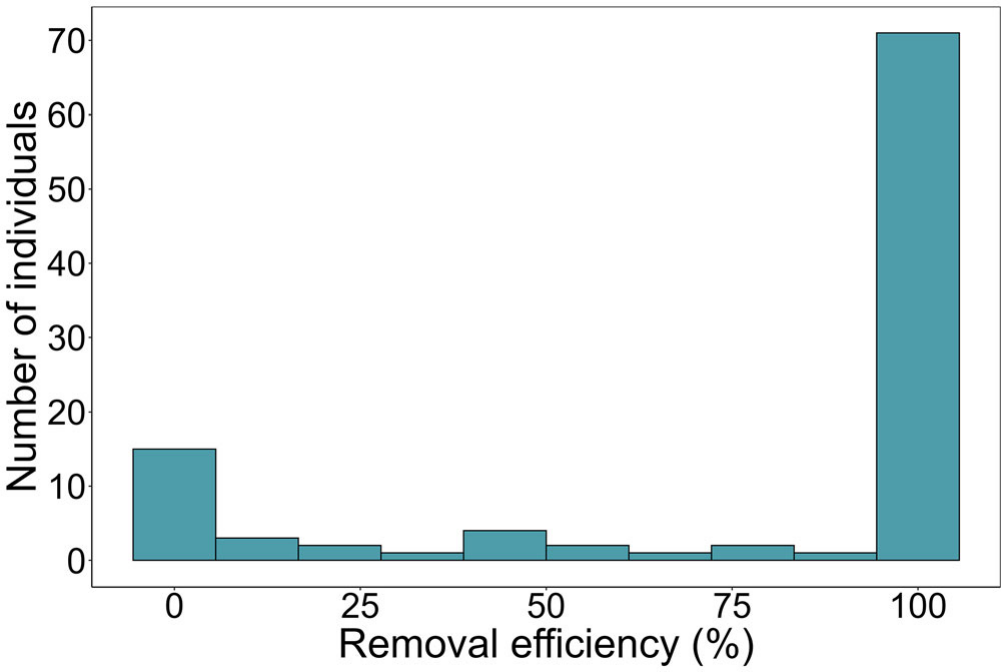
Histogram of removal efficiency of each individual *Anguilla marmorata* eels.

**FIGURE 5:**
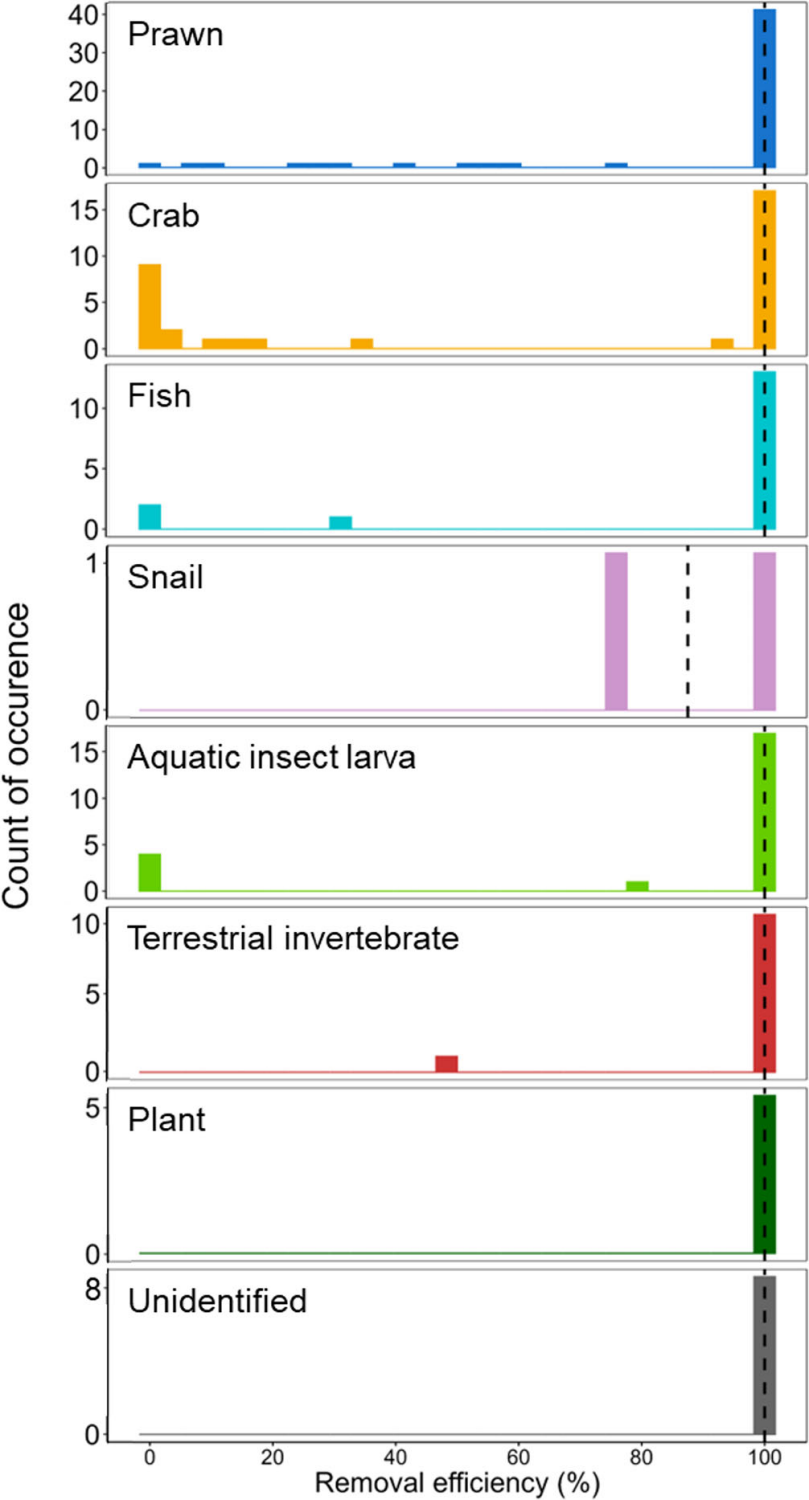
Histogram of removal efficiency of each individual *Anguilla marmorata* eel for each prey category. Vertical dashed lines indicate the median removal efficiency for each prey category.

The removal efficiency of the tube method for each individual was 76.5% ± 38.9% (mean ± SD), which showed a bimodal distribution (Silverman's test, *p* > 0.05), with peaks at 0% and 100% (Figure 4). All stomach contents were collected from 66.7% of individuals (68 out of 102 individuals) using the tube method, whereas no stomach contents were collected from 11.8% of individuals (12 out of 102 individuals). For each prey category, the removal efficiency of the tube method was higher than 80% for most categories, ranging from 56.7% to 100%, with crabs showing the lowest efficiency (Figure 5). The detection rate for each prey category was 92.4% ± 10.8% (mean ± SD), ranging from 71.9% to 100% (Table 1). The detection rate for crab was the lowest (70%) in the prey categories.

**TABLE 1:** Detection rate for each prey category from 102 *Anguilla marmorata* having stomach contents.

Prey category	Count of occurrences		Detection rate
	Tube	Dissection only	
Prawn	48	1	98.0
Crab	23	9	71.9
Fish	14	2	87.5
Snail	2	0	100.0
Aquatic insect larva	18	4	81.8
Terrestrial invertebrate	10	0	100.0
Plant	5	0	100.0
Unidentified	9	0	100.0

*Note*: Detection rate was calculated as the ratio of the number of occurrences of each prey category identified using the tube method to those identified only through dissection.

The full model showed that all explanatory variables did not significantly affect the removal efficiency of stomach contents (Table S2). The best‐fitted model for the removal efficiency included none of the explanatory variables (i.e., null model) (Table 2). No significant difference was found between the composition of prey categories by the tube method and that collected using both the tube method and dissection (PERMANOVA, *F* = 1.168, *p* > 0.05, *R* ^*2*^ = 0.293), indicating that the stomach content composition collected by the tube method can reflect the entire stomach content.

**TABLE 2:** Akaike's Information Criterion (AIC) ranking of the generalised linear model (GLM) that explains removal efficiency using the tube method for *Anguilla marmorata*.

Rank	Explanatory variable estimates			AIC	ΔAIC
	Prey category	Stomach fullness	Total length		
1				114.523	0
2			0.001	115.589	0.066
3		0.038	0.002	116.038	1.515
4		0.025		116.317	1.794
5	+			116.36	1.837

*Note*: Plus sign and no symbol indicate significant or no significant effects of qualitative variables on the removal efficiency, respectively. Only models with ΔAIC <2 are shown.

#### 3.1.2 Effectiveness of the forceps method

In the stomach contents of 12 individuals for which no stomach contents were collected using the tube method, crabs were found from seven eels (58.3% of eels), aquatic insect larvae were found from four eels (33.3%) and fish and prawns were found from one individual (8.3%) through dissection. To improve the removal efficiency of the tube method for such individuals, additional sampling was performed. As a result, four individuals were captured in October 2024 to evaluate the efficiency of using the forceps method following the tube method process. The total length and body weight of these eels were 349, 498, 530 and 630 mm, and 87, 381, 349 and 591 g, respectively. The removal efficiency using both the tube and forceps methods was 100% for all individuals, all of which fed on crab or prawn. Although the stomach contents of two individuals were obtained using both the tube and forceps methods, those of the remaining two individuals were successfully collected using only forceps.

### 3.2 Stomach contents of *A. marmorata*

**FIGURE 6:**
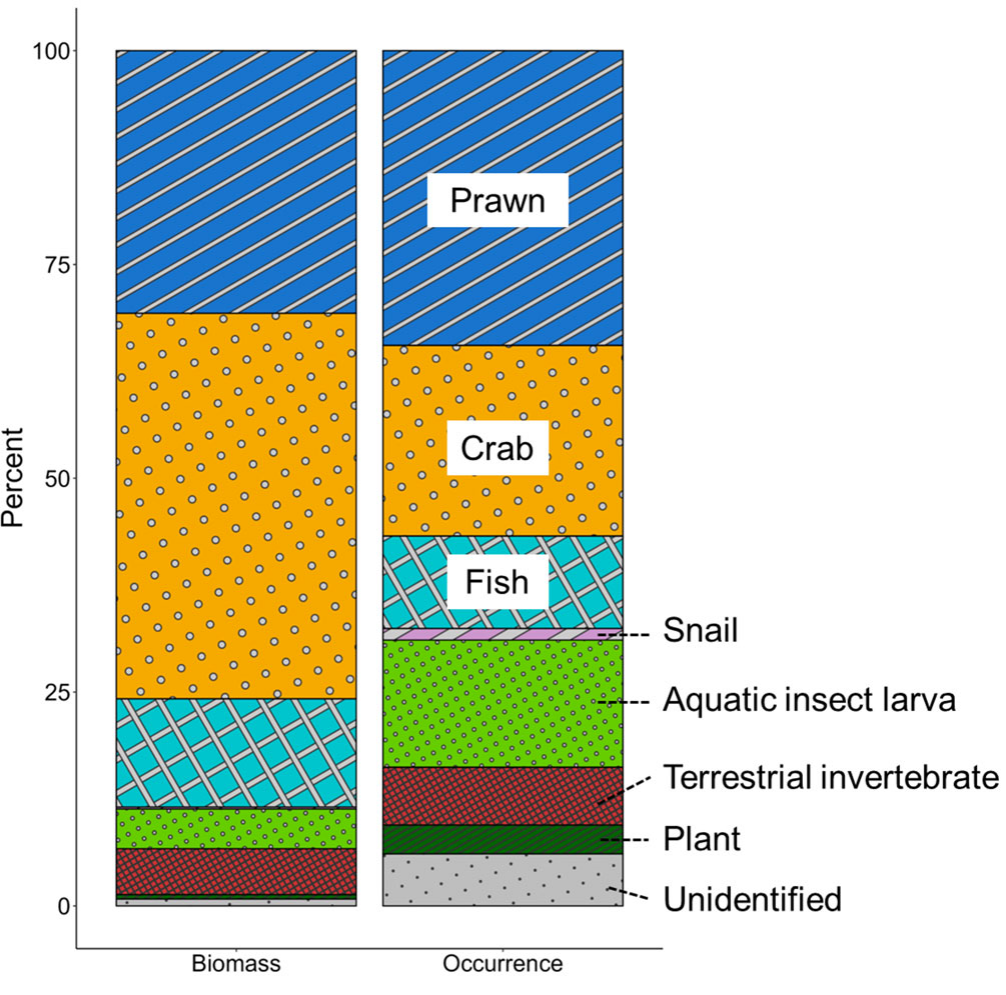
Types of food items in the stomach contents of *Anguilla marmorata* on Amami–Oshima Island. The left ratio indicates the stomach contents in terms of biomass, and the right ratio indicates in terms of occurrence.

The most frequently observed prey items in the stomach contents of *A. marmorata* were crustaceans (Figure 6). In particular, 46.1% of individuals contained *Macrobrachium* prawns in their stomach contents. (Table S3). Crustaceans (prawns and crabs) comprised more than 50% of both biomass and occurrence in stomach content composition, which were followed by fish, aquatic insect larvae and terrestrial invertebrates. A total of 36 individuals (34.0%) had more than one prey category. *A. marmorata* fed on various types of aquatic and terrestrial animals, which included five families of crustaceans (one order), two families of fish (one order), two families of snails (one order), four orders of aquatic insect larvae, four orders of terrestrial invertebrates and two families of plants (two orders). The crabs included the families Potamidae, Sesarmidae and Varunidae, with the species *Geothelphusa sakamotoana*, *Chiromantes dehaani*, *Eriocheir japonica* and *Ptychognathus ishii* being identified. The prawns included the families *Palaemonidae* and *Atyidae*, which included *Macrobrachium formosense*, *Macrobrachium japonicum* and *Caridina serratirostris*. Fish included the family *Gobiidae*, *Eleotridae*, with *Luciogobius* spp., *Rhinogobius nagoyae*, *Sicyopterus japonicus* and *Eleotris fusca* being identified. Snails included the families *Neritidae* and *Septariidae*, which included *Clithon retropictus* and *Septaria lineata*. The aquatic insect larvae included *Ephemeroptera*, *Trichoptera*, *Megaloptera* and *Diptera*, with larvae of *Stenopsyche marmorata* and *Protohermes* spp. being found. Terrestrial invertebrates included *Orthoptera*, *Blattaria*, *Hemiptera* and *Araneae*, with *Pyrgocorypha subulate* and *Rhabdoblatta* spp. being distinguished. The plants included *Poaceae* and *Polygonaceae*.

## 4 DISCUSSION

### 4.1 Efficiency of non‐lethal stomach content analysis

This study evaluated the effectiveness of the tube method as a non‐lethal stomach content analysis technique for *A. marmorata*, and it demonstrated that it achieved a removal efficiency of 76.5% and a prey category detection rate of 92.4%. The removal efficiency is comparable to, or in some cases higher than, the values reported in previous studies on other fish species, including largemouth bass *Micropterus salmoides floridanus* (80%; Cailteux et al., 1990), butterfly peacock bass *Cichla ocellaris* (57%; Bies & Neal, 2016) and walleye *Stizostedion vitreum* (63%; Quist et al., 2002), using the tube method, and *A. rostrata* (89%) using the gastric lavage method (Studio & May, 2025). The stomach content composition collected by the tube method did not significantly differ from that collected using both the tube method and dissection. This indicates that the stomach content composition collected using the tube method accurately reflects the feeding habits of eels, which is conventionally studied using the lethal method. Moreover, the best‐fitted GLM did not include any explanatory variables considered in this study, suggesting that the tube method is effective for collecting stomach contents of eels regardless of body size, stomach fullness or prey category. This study used six tube sizes appropriate for gape size, which may have contributed to this effectiveness. Using the largest‐diameter tube possible can help maximise stomach content collection (Van Den Avyle & Roussel, 1980). Therefore, the tube method can be a useful tool for investigating the feeding habits of *A. marmorata*, although it should be noted that the removal efficiency is unclear for individuals <150 mm and >1100 mm, both of which were not assessed in this study.

The removal efficiency for the collection of stomach contents of fish can potentially be improved further by using the forceps method after the tube method. In this study, the removal efficiency of the tube method showed a bimodal distribution, indicating that individuals were broadly classified into those from which all stomach contents were collected (a peak at 100%) and those from which none were collected (another peak at 0%). This pattern is similar to that observed in the previous study on *C. ocellaris* (Bies & Neal, 2016), where individuals were divided into those whose stomach contents were completely collected and those in which all contents remained. Thus, a key factor in further improving the removal efficiency of the tube method is to make the remaining stomach contents in individuals with 0% removal efficiency retrievable. In this study, the stomach contents that remained in individuals from which no stomach contents were removed were composed of crustaceans (crabs and prawns), aquatic insect larvae and fish. In particular, removal efficiency for crabs was the lowest among all prey species (Figure 5), likely because the body size of large crabs is greater than the inner diameter of the tube, or because their hard exoskeleton and projections may get stuck in the stomach wall, preventing removal. A similar observation has been reported for *A. rostrata*, where crustaceans were found to be less likely to be collected from stomach contents using the gastric lavage method (Studio & May, 2025). Our study effectively overcame these low removal efficiencies associated with non‐lethal methods, demonstrating that using the forceps method after the tube method enables the complete collection of food items such as crabs and prawns by directly grasping them with forceps, highlighting its effectiveness in retrieving large prey items. Therefore, combining the tube and forceps methods is likely to be a powerful approach for accurately collecting eel stomach contents, leading to a more precise assessment of their feeding habits.

The tube method is considered unlikely to cause serious injury to eels. Our study confirmed that both tube and forceps methods did not cause stomach wall injury in *A. marmorata*. Stomach damage due to spiny rays and pressure applied to the abdomen has been reported in *C. ocellaris* (Bies & Neal, 2016), but no such damage was observed in this study, probably because prey species with strong spines were not identified from stomach contents of the target species. Stomach content collection in this study took 3–5 min per individual, and no mortality or immediate behavioural abnormalities were observed. All individuals fully recovered from anaesthesia immediately following all handling procedures. Therefore, this non‐lethal method might enable the repeated collection of eel stomach contents over the long term without damaging individuals and the population, providing several advantages over conventional approaches. However, long‐term survival monitoring is necessary to confirm if stomach contents can indeed be repeatedly collected using this method in combination with individual identification markers (Myers et al., 2025).

### 4.2 Feeding habits of *A. marmorata*

*A. marmorata* consumes a wide range of aquatic and terrestrial organisms, similar to other anguillid eels, such as *Anguilla japonica* (Itakura et al., 2015; Wakiya & Mochioka, 2021), *Anguilla australis* and *Anguilla dieffenbachii* (Jellyman, 1989). Crustaceans were the primary prey item, a pattern consistent with previous studies on other anguillid eels (Romanda et al., 2019). Few individuals contained plants, which might have been accidentally ingested by the eels, as anguillid eels are generally considered to be carnivores (Jellyman, 1989). In contrast, *A. marmorata* on the study island tended to prey on multiple prey types within short durations (e.g., per 1–2 days), with 34% of individuals having more than one type of prey. This is somewhat different from those reported for 3.1% in *A. japonica* (Kaifu, Miller, et al., 2013) and 1.9% in *Anguilla anguilla* (Dorner et al., 2009). Most of the *A. japonica* and *A. anguilla* eels studied fed on one type of prey at each feeding event, whereas *A. marmorata* on the study island may be more opportunistic and feed on any available prey. Whether this difference in feeding strategy is due to species‐specific traits or environmental factors remains unclear, and further research is needed.

However, the gut contents found in our study are also a reflection of the types of species that are found in tropical island rivers, which include many diadromous species such as amphidromous prawns and gobies (reviewed by Miller et al., 2024). For example, the main prey item of *A. marmorata* at Tahiti and Moorea in the South Pacific region was *Macrobrachium* prawns, but amphidromous gobies were also important (Marquet & Lamarque, 1986). A few individuals contained plants, which might have been accidentally ingested by the eels, as anguillid eels are generally considered to be carnivores (Jellyman, 1989). Unlike the rivers in our study, three species of tropical anguillid eels are present in the South Pacific, which appear to have different but overlapping diets (Marquet & Lamarque, 1986). Using the tube method to examine their diets would enable new studies to be conducted to better understand the feeding ecologies of those sympatric species, without harming their populations.

As observed in its diverse stomach contents, *A. marmorata* is the largest top predator in the riverine ecosystems of the study island, where they interact with the populations of numerous aquatic organisms. A top predator may regulate populations through top‐down control, triggering a trophic cascade and influencing interspecific interactions within the ecosystem (Sergio et al., 2008). Understanding the feeding habits of *A. marmorata* may contribute to unveiling the complex food web and trophic dynamics in tropical and subtropical rivers, such as Amami–Oshima Island, where there are few other large predatory fishes. This type of non‐lethal method may be particularly useful for tropical eels, especially in regions such as tropical islands where their populations are smaller, as in the present study.

Non‐lethal stomach content analysis of anguillid eels may provide detailed knowledge of feeding ecology at the individual level. Individual diet specialisation and plasticity can be demonstrated by repeated surveys using a non‐lethal method combined with individual identification tags (Iguchi et al., 2004). Moreover, combining this approach with other techniques, such as stable isotope analysis and DNA barcoding of gut contents, could provide a more detailed and comprehensive understanding of their dietary composition (Denis et al., 2022; Guillerault et al., 2017). Although lethal stomach content analysis showed that anguillid eels consume a single type of prey item per feeding event (Kaifu, Miyazaki, et al., 2013), suggesting diet specialisation, nothing is known about how this individual feeding tactic will change over time. Combining tagging with the tube method established in this study would allow continuous monitoring of anguillid eel feeding habits at the individual level, revealing individual dietary variations, including seasonal changes and dietary plasticity. With eel populations declining, the method we evaluated can provide important information for the management of eel stocks and riverine ecosystems, while reducing the impact of research on their population size.

## AUTHOR CONTRIBUTIONS

Tatsuhiko Maeda performed the field data collection and arranged the sampling in the field. Tatsuhiko Maeda performed the analysis with help from Hikaru Itakura, Ryoshiro Wakiya and Shingo Kimura. The manuscript was written by Tatsuhiko Maeda and Hikaru Itakura. All authors contributed and approved the final version of the manuscript.

## FUNDING INFORMATION

This study was partly supported by the River Fund of the River Foundation, Japan (grant number: 2024‐5311‐008), Japan Science and Technology Agency (grant number: JPMJSP2108) and JSPS KAKENHI (grant number: 21H04939).

## CONFLICT OF INTEREST STATEMENT

The authors declare no conflicts of interest.

## Supporting information


**Data S1.** Supporting information.

**Video S1.** Demonstration of tube and forceps methods for non‐lethal stomach content analysis of *Anguilla marmorata*.

## Data Availability

All data generated or analysed in this study are included in this article. Any additional data used or analysed during this study are available from the corresponding author upon reasonable request.
