# Supplementary material for: Development of a non‐lethal stomach content analysis method for freshwater eels: An empirical evaluation of the tube method for Anguilla marmorata
Source: J Fish Biol. 2025 Aug 27;107(6):1995–2004. doi: 10.1111/jfb.70198 (PMC12861835; doi:10.1111/jfb.70198)
Supplement: Supplementary file 1 — Data S1. Supporting information. [file JFB-107-1995-s002.docx]

**Supplemental Information**

**Development of a non**–**lethal stomach content analysis method for freshwater eels: An empirical evaluation of the tube method for *Anguilla marmorata.***

Tatsuhiko Maeda^1^ | Hikaru Itakura^1^ | Ryoshiro Wakiya^1^ | Shingo Kimura^1^

^1^Atmosphere and Ocean Research Institute, The University of Tokyo, 5-1-5 Kashiwanoha, Kashiwa, Chiba, 277-8564, Japan

Corresponding Author: hikaruitakura@aori.u-tokyo.ac.jp

**List of contents**

**1) Supplemental Table**

Table S1. Sampling year-month, river and number of captured individuals.

Table S2. Full model summary of the generalised linear model (GLM).

Table S3. Frequency-based diet of *Anguilla marmorata.*

**2) Supplemental Video**

Video 1 Demonstration of non-lethal stomach content analysis.

**TABLE S1** Sampling year-month, river and number of captured individuals.

**
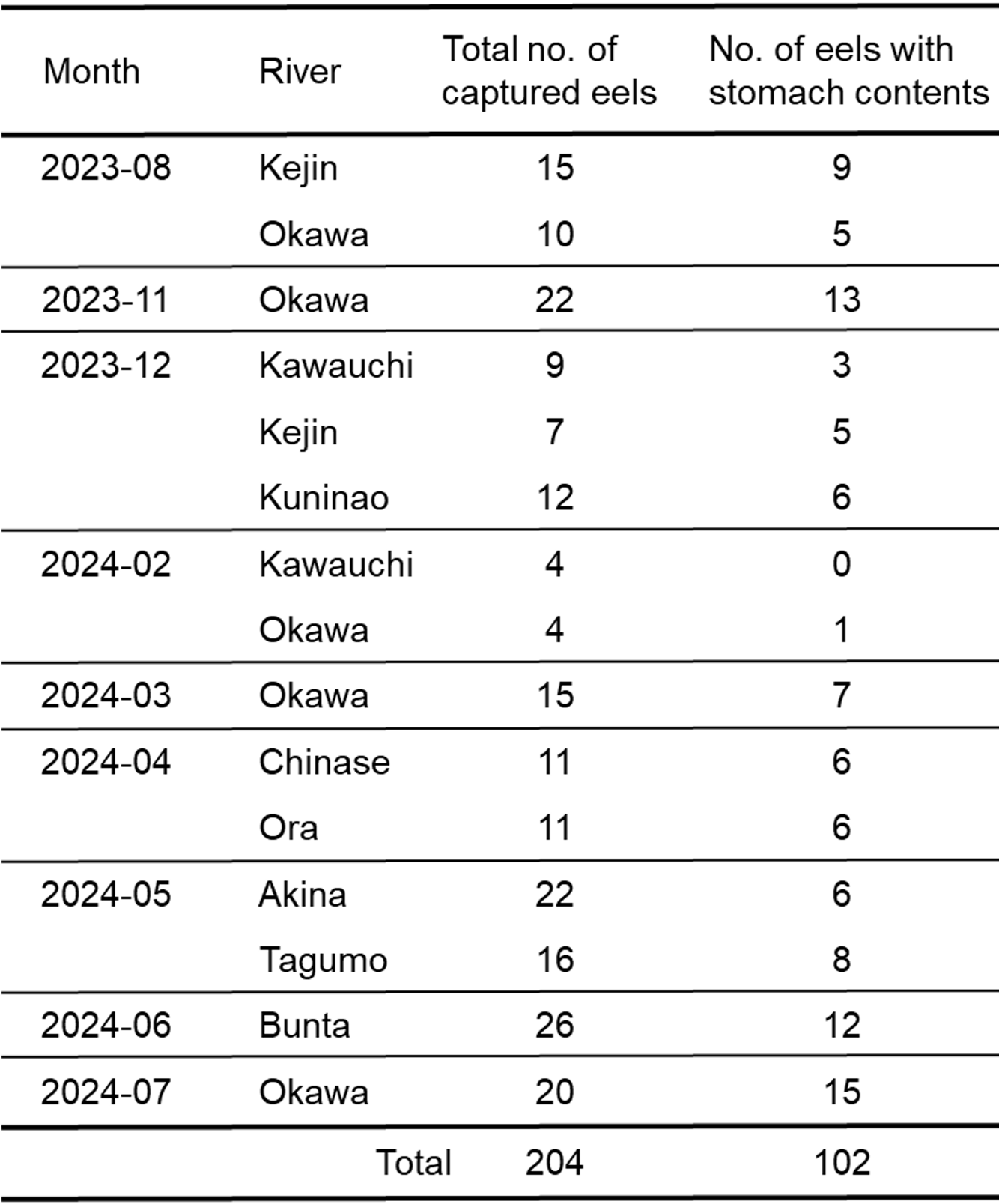
**

**TABLE S2** Full model summary of the generalised linear model (GLM) examining factors influencing removal efficiency.

**
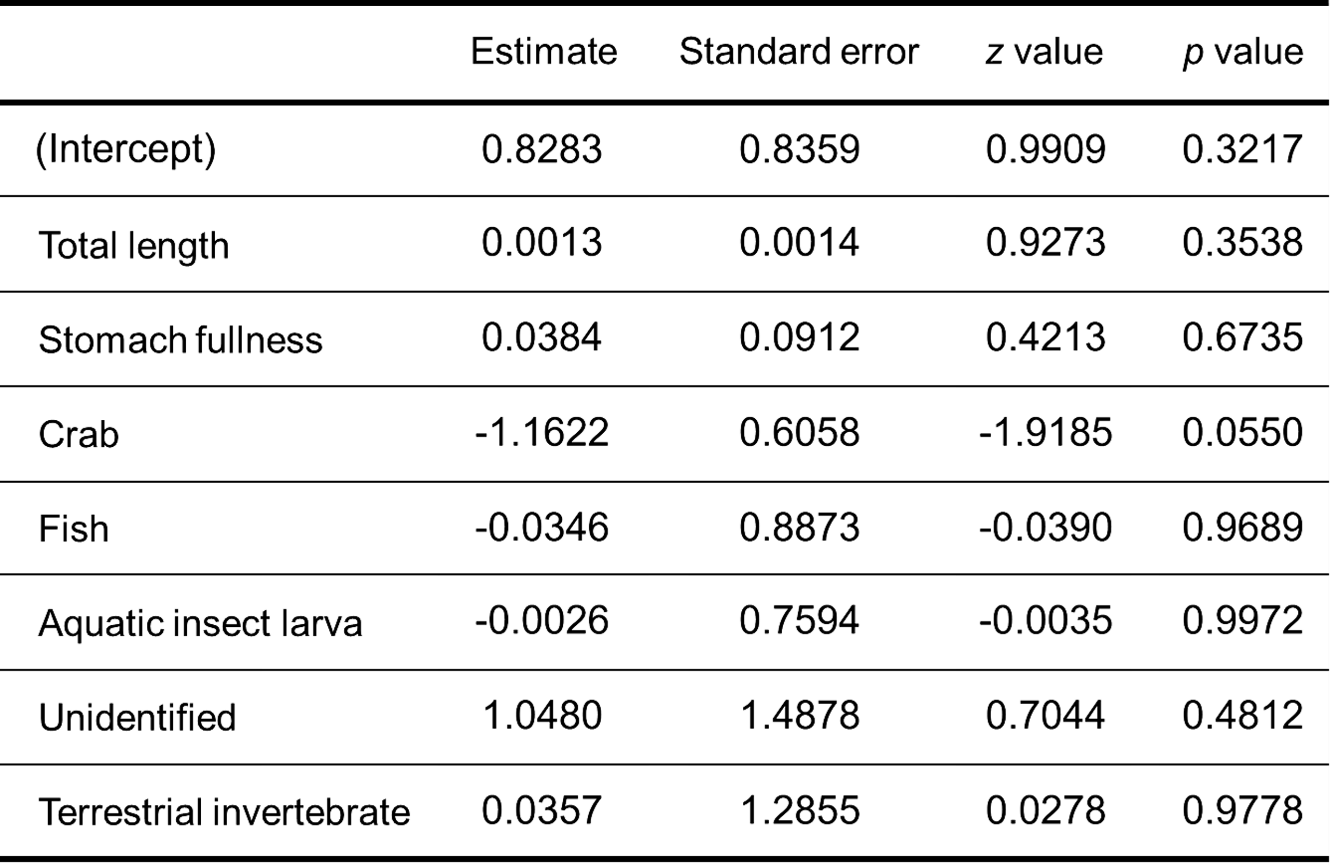
**

**TABLE S3**Diet of *Anguilla marmorata* on Amami-Oshima Island, based on percent of frequency of occurrence (%F).

**
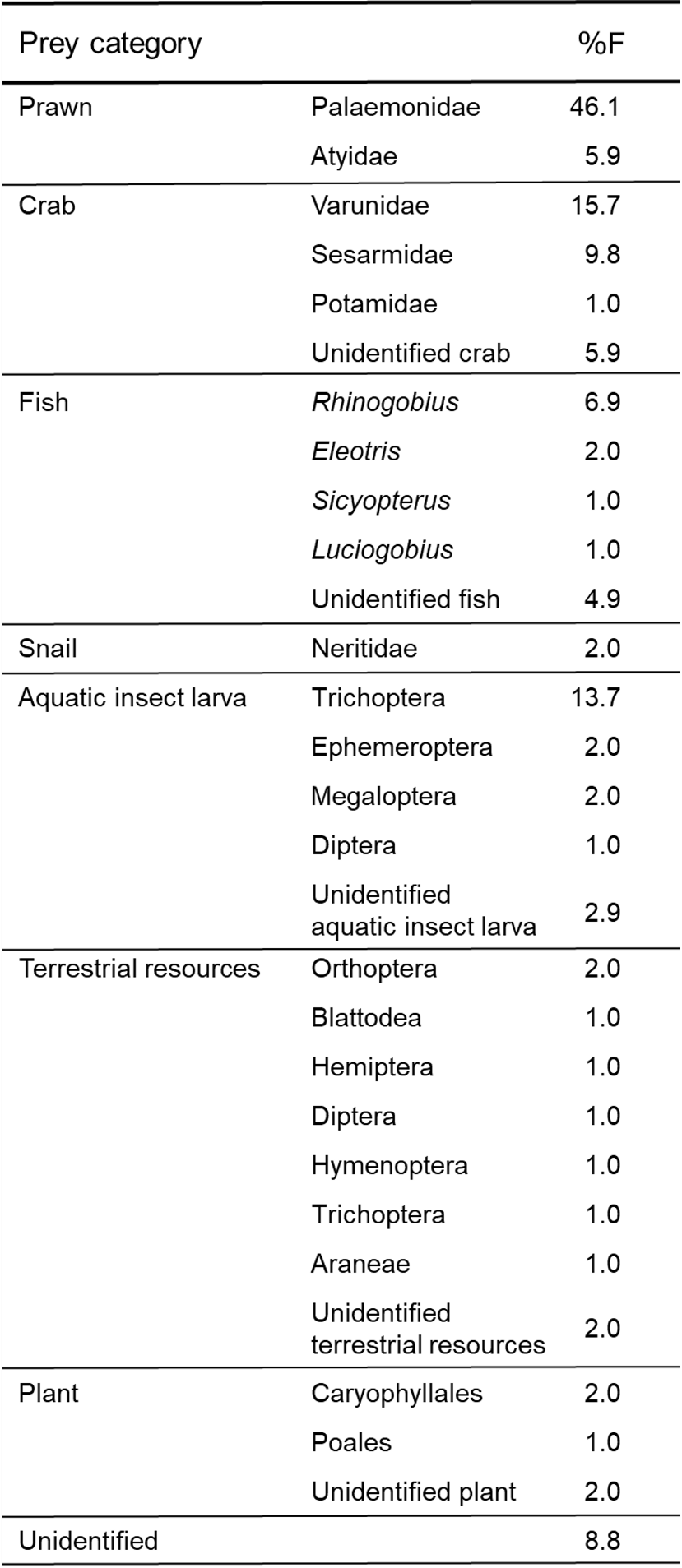
**

**VIDEO 1** Demonstration of tube and forceps methods for non-lethal stomach content analysis of *Anguilla marmorata*.
